# Strong evidence for age as the single most dominant predictor of medically supervised driving test—mini mental status test outcomes provide only weak but significant moderate additional predictive value

**DOI:** 10.1186/s12877-022-02951-6

**Published:** 2022-03-24

**Authors:** Yannik Isler, Simon Schwab, Regula Wick, Stefan Lakämper

**Affiliations:** 1 Institute for Forensic Medicine, Traffic Medicine, University of Zürich, Andreasstrasse 15, 8050 Zürich, Switzerland; 2 Center for Reproducible Science, University of Zürich, Hirschengraben 84, 8001 Zürich, Switzerland; 3 Epidemiology, Biostatistics and Prevention Institute, Hirschengraben 84, 8001 Zürich, Switzerland

**Keywords:** Fitness to drive, Traffic medicine, Elderly drivers, Cognitive testing, Medically supervised driving test, Mini mental status test, Clock test, Trail making test, Receiver operating characteristics

## Abstract

**Background:**

With age, medical conditions impairing safe driving accumulate. Consequently, the risk of accidents increases. To mitigate this risk, Swiss law requires biannual assessments of the fitness to drive of elderly drivers. Drivers may prove their cognitive and physical capacity for safe driving in a medically supervised driving test (MSDT) when borderline cases, as indicated by low performance in a set of four cognitive tests, including e.g. the mini mental status test (MMST). Any prognostic, rather than indicative, relations for MSDT outcomes have neither been confirmed nor falsified so far. In order to avoid use of unsubstantiated rules of thumb, we here evaluate the predictive value for MSDT outcomes of the outcomes of the standard set of four cognitive tests, used in Swiss traffic medicine examinations.

**Methods:**

We present descriptive information on age, gender and cognitive pretesting results of all MSDTs recorded in our case database from 2017 to 2019. Based on these retrospective cohort data, we used logistic regression to predict the binary outcome MSDT. An exploratory analysis used all available data (model 1). Based on the Akaike Information Criterion (AIC), we then established a model including variables age and MMST (model 2). To evaluate the predictive value of the four cognitive assessments, model 3 included cognitive test outcomes only. Receiver operating characteristics (ROC) and area under the curve (AUC) allowed evaluating discriminative performance of the three different models using independent validation data.

**Results:**

Using *N* = 188 complete data sets of a total of 225 included cases, AIC identified age (*p* < 0.0008) and MMST (*p* = 0.024) as dominating predictors for MSDT outcomes with a median AUC of 0.71 (95%-CI 0.57–0.85) across different training and validation splits, while using the four cognitive test results exclusively yielded a median AUC of 0.55 (95%-CI 0.40–0.71).

**Conclusions:**

Our analysis provided strong evidence for age as the single most dominant predictor of MSDT outcomes. Adding MMST provides only weak additional predictive value for MSDT outcomes. Combining the results of four cognitive test used as standard screen in Swiss traffic medicine alone, proved to be of poor predictive value. This highlights the importance of MSDTs for balancing between the mitigation of risks by and the right to drive for the elderly.

## Background

Within the last five decades (1970–2019) the number of fatalities in Swiss traffic has decreased by about 90% [1]. In 2019, Swiss authorities reported a record low number of 187 fatalities in traffic. The number of accidents with severe personal damage has decreased in all age groups, except in that of the elderly, i.e. drivers aged > 65. Elderly drivers are responsible for 10% of severe accidents and two out of three passenger car collisions are caused by senior drivers [1]. These figures relate to an increase in physical and cognitive performance deficits with age. These deficits might be due to age degeneration per se, to the life-long accumulation of medical events, or to medical conditions strongly associated to age, such as dementia, Alzheimer’s disease or diabetes, [2–4].

To mitigate the risk in traffic resulting from age related performance deficits, Swiss law [5] obliges all license holders to fulfill medical minimum requirements (MMR) as further detailed in ordinances [6] and guidelines [7]. MMRs cover all medical aspects relevant for driving, for example vision, somatic and psychiatric conditions, possible substance abuse and general cognitive performance. MMRs are controlled by experts in traffic medicine, who are organized in a four-tier system, ranging from a trained physician (level 1) to full-time experts in traffic medicine (level 4).

For elderly drivers above age 75, mandatory biannual checkups and control of MMRs ensure their fitness to drive (FTD), which is defined as the general and non-transient physical and mental aptitude to safely conduct a vehicle in traffic. Apart from the patient’s general medical status and history, an assessment of the FTD takes into account the general performance capabilities, assuming that safe driving requires both a basic physical and mental capacity for undisturbed traffic as well as a mental and physical reserve capacity, relevant in unforeseen situations [8].

Usually, these biannual FTD-assessments of elderly drivers are performed by a family doctor, trained at level 1. Increasing deficits may allow calling for a more comprehensive assessment at level 3 or level 4 experts. Here, a global medical examination and assessment of all medical records should ensure meeting all MMRs. If even results of a level 4 examination is not sufficiently conclusive to decide on the drivers FTD, Swiss law provides the opportunity for the assessing expert to offer an on-road driving test, the medically supervised driving test, MSDT. An MSDT is performed in the driver’s own car and in presence of both an experienced representative of the authority issuing the driving license (RA) and a traffic medicine expert (TME), typically level 4. Similar to a driving exam, the RA provides the driver with verbal instructions about the route, which is not fully standardized but rather adapted depending on traffic, to provide sufficient critical situations for both RA and TME to evaluate the driver’s abilities. Evaluation of the MSDT follows a non-itemized and verbal scoring of broadly defined dimensions, such as the ability to safely and routinely control the vehicle, to adapt to changing traffic, the ability to follow directions/instructions, and driving errors, such as missed lights, speeding, near collisions, incomplete stops, and alike.

Current guidelines indicating this medically supervised driving test (MSDT) are not fully harmonized. However, the decision to offer an MSDT is usually, but not exclusively, based on indications of cognitive deficits to some degree. In a typical level 4 assessment of the FTD, such presumed or previously recorded cognitive deficits are always re-assessed briefly by way of a standard set of four standard cognitive test, i.e. the mini-mental status exam or test (MMST) [9], the clock test (CT) [10], and part A and B of the trail-making test (TMT-A and TMT-B) [11, 12].

Execution and evaluation of these four cognitive tests follows published guidelines [8]. Accordingly, scoring less than 24 of the maximal 30 points in the MMST indicates an increased likelihood of a mild dementia and a lowered ability to drive safely [13]. Scoring between 24–27 points might indicate an increased likelihood of mild cognitive impairment (MCI) [14]. Similarly, a result of six or more points of the maximal seven points in the clock-test (CT) is considered normal. However, literature reports zero to three errors to be normal, with an error score higher than four having a sensitivity of 82% and a Cohens Kappa for interrater reliability of 0.7 for identifying dementia as referred in [15, 16]. A less permissive error-score of only up to two in the CT might encompass conditions preceding dementia such as mild-cognitive impairment (MCI). The trail-making tests A and B are assessed using condensed stratification by age, sex and education [7]. More detailed stratification data is available elsewhere [17].

The selected set of tests is the usual—but by no means exclusive—tool to indicate for an MSDT. Time and “experience” has inevitably led to the impression that simplified “rules of thumb” based on the results might also be able to predict MSDT-outcomes. Such unsubstantiated – but also insufficiently falsified – claims persist in practice. For example, several practical, but subjective guidelines [18, 19] state that elderly drivers with MMST test-values < 21 or a TMT-B-value of > 180 (independent of age) are highly unlikely to pass an MSDT [8].

Based on a retrospective sample set, we aim to evaluate systematically the predictive value of age, gender, and the results of the above-mentioned four standard cognitive tests for MSDT outcomes. Using data of all performed MSDTs within a three-year period, we develop, validate and compare multivariable prediction models and their respective predictive value for MSDT outcomes. Using this rigorous approach on retrieved retrospective data, we hope to contribute to settling the issue whether cognitive tests alone, single or combined, might predict MSDT outcomes with acceptable accuracy.

### Materials and methods

Overall, the manuscript was prepared following the TRIPOD checklist for model prediction development and validation. From our in-house records, we retrospectively analyzed data related to all MSDTs performed within the years 2017–2019. For this project, we primarily extracted MSDT outcome, age at MSDT, gender, and results of the four cognitive standard tests (MMST, CU, TMT-A and TMT-B) acquired in house prior to the decision to offer an MSDT (typically 6–8 weeks). Additional, secondary data were collected within the scope of YI’s thesis. Of these, categorized data of indication leading to an MSDT were integrated in the results and discussion. Driving experience, educational level, driving exposure and accident history were not recorded and correlated. Data was collected in Excel according to a codebook. The acquired data was then analyzed using R (version 4.0.2).

For data acquisition, we mined two partially overlapping databases of the Traffic Medicine at the Institute of Forensic Medicine of the University of Zurich (TMZ): a.) LOTUS, a case-based system allowing limited key-word searches of selected documents including the final assessment of the fitness to drive and b.) Docuware, a fully OCR-searchable document archive of the complete patient record as obtained in the context of assessment at TMZ. Generally, in Lotus, the MSDTs itself and its protocol was listed as a separate, additional entry, independent of prior external or in-house assessment leading to an actual MSDT. The desired information concerning cognitive testing and patient history either from in-house or outside experts was thus gathered from these separate database entries in Lotus. Wherever possible, the information was complemented using the full patient records in Docuware if information was lacking in Lotus entries, or vice versa. As TMZ is one of the few institutions providing MSDT-based assessments in Switzerland, cases from drivers within and outside of the canton of Zürich were registered. Known inconsistencies in entering information into the database Lotus prior to 2019 led to a separate, manual list of all MSDTs performed at TMZ starting from 2017 in Excel. For this work, cases and all associated databased entries were consequently manually congregated from this list. Fundamental demographic data, cognitive test results, indications for an MSDT as well as protocols of MSDTs were obtained. Data was collected in Excel according to a codebook.

#### Inclusion/Exclusion

According to the manual records, 243 MSDTs were offered between January 1, 2017, and December 31, 2019, as advised for by preceding examinations in traffic medicine. Eleven drivers did not attend the appointment or handed in their driving license to the authorities prior to the appointment and were thus excluded from the analysis. Four drivers were incorrectly allowed to perform a second MSDT. These cases were excluded, as duplicate MSDTs are not foreseen by law and regulations. Consequently, a total of 225 MSDT-cases and associated data for indications and cognitive tests were included in the descriptive part of this study.

#### Statistical analysis

For our multivariable prediction model development and validation we used *N* = 188 complete cases; thus 37 (16%) of the patients were excluded (28 missings in MMST; 27 UT; 20 TMTA and 20 TMTB). After checking for variable collinearity, we randomly split the data for training (2/3) and subsequent validation (1/3). We thus used a fixed sample size (complete cases). Therefore, instead of the required sample size, we determined the required events per parameter (EPP) for the full model that included six variables (age, sex, MMST, UT, TMTA, TMTB). This resulted in 125 events and 63 events per 6 parameters in the training and validation data, respectively (EPP_Train_ = 20.8, EPP_valid_ = 10.5). A rule of thumb is to have at least 10 events per parameter.

Class distribution in the complete data was MSDT failed 77 (41%) vs. passed 111 (59%). Balancing the training sub-set by synthetic minority oversampling technique (SMOTE) addressed the common bias toward the majority class. Receiver operating characteristics (ROC) and area under the curve (AUC) allowed evaluating discrimination performance of the different models using unseen validation data. We additionally calculated results after *k* = 2,000 iterations across randomly split data into different training and validation sets providing a median AUC and related confidence interval.

## Results

### Indications, demographic information and overall MSDT results

Out of six indication groups (in short: cognitive, somatic, psychiatric, incident, substance, traffic psychology) for an MSDT the three most frequently recorded were a.) Cognitive deficits (*N* = 184), b.) Somatic conditions (*N* = 87) and c.) Incidents in traffic (*N* = 33). Multiple indications were allowed to be recorded (although limited to a maximum of three), resulting in 316 entries. Based on this, the indication “cognitive deficits” alone accounted for 58% of all counted indications and was listed in 82% of the 225 cases. The three above mentioned indications alone accounted for 96% of all indications, the sum of the remaining indications (i.e. incident, substance, traffic psychology) represents only 4% of MSDTs, respectively (*N* = 12).

**Fig. 1:**
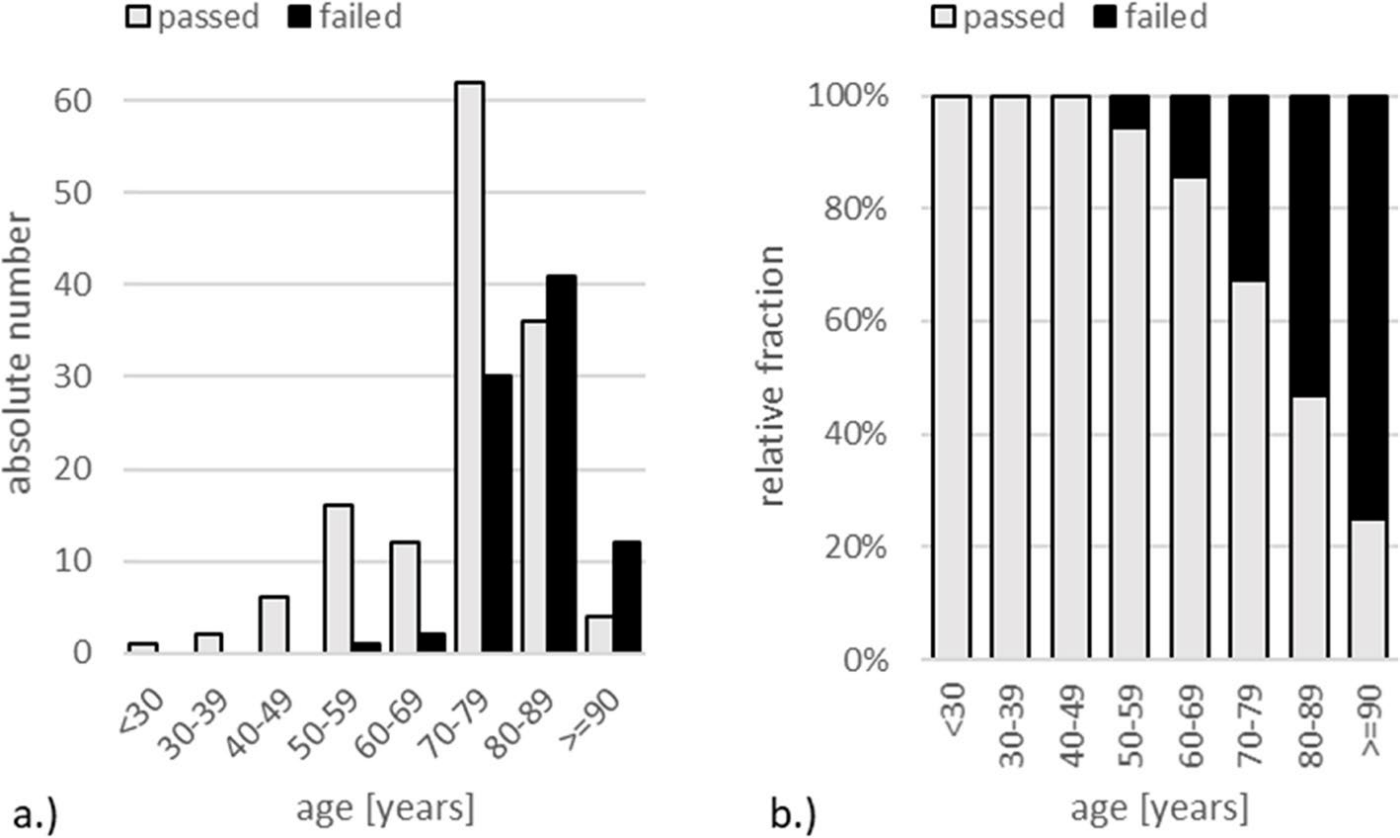
**a** Absolute and **b** relative MSDT results by 10-year age brackets

MSDTs were taken by 179 (80%) male and 46 (20%) female drivers. The average age at the time of MSDT was 75 years, with a span ranging from 25 to 93 years of age. 75% of all cases were aged 70 and above at the MSDT. 38% (*N* = 86) failed in the MSDT (Fig. 1a.). When grouping MSDT results in 10-year age brackets, the fraction of drivers failing the MSDT steeply increases at ages 70 and higher (Fig. 1b).

### Cognitive testing results

The decision to allow for an MSDT is often, but not exclusively, based on indications of cognitive deficits. Whether already documented or not, a level 4 assessments of elderly drivers usually include four cognitive tests, i.e. the MMT, CT, TMT-A and TMT-B. With respect to these four test, our overall cohort of 225 MSDT cases was incomplete. The full set of cognitive tests was recorded in 84% (*N* = 188) of all cases. For 5% (*N* = 11), 4% (*N* = 10) and 7% (*N* = 16) of all cases data for one, two or four cognitive tests, respectively, were not to be found in the records.

Of the four cognitive tests, TMT-A and TMT-B were recorded for 91% of all MSDT cases (i.e. data are missing for 20 of 225 cases), while both MMST and CT were recorded for only 84% of all MSDT cases (i.e. data are missing for 28 or 27 cases, respectively).

Patients failed in 5% of MMST- (< 27 points), 18% of CT- (< 5 points), 37% of TMT-A- and 60% of TMT-B-tests actually taken. 21% (*N* = 44) of the patients could not terminate the TMT-B-test and gave up.

The average test result in the MMST was 27.4 points out of 30. 27 drivers obtained a maximum of 30 points. The lowest recorded value was 15 (*N* = 1). The average test result in the CT was 5.8 points out of 7. 109 drivers obtained a maximum of 7 points. 5 drivers scored 0 points.

### Comparing cognitive tests results and MSDT-outcome

The 116 MMST-scores of drivers who passed the MSDT averaged to 27.6 of 30 points, while the 81 MMST-scores of those who failed the MSDT averaged to 27.1 of 20 points. Similarly, the CT-scores averaged to 5.9 and 5.4 of 7 point for the 117 drivers who passed and the 81 drivers who failed the MSDT 81 (64%) of the 129 drivers with TMT-A test-times within the norm (“passed”) and 44 (58%) of the 76 drivers with TMT-A test-times exceeding the norm (“failed”) were able to pass the MSDT..

Similarly, 55 (65%) of the 85 drivers with TMT-B test-times within the norm (“passed”) and 67 (55%) of the 122 drivers with TMT-B test-times exceeding the norm (“failed”) were able to pass the MSDT. Of the 44 of 205 drivers who could not finish the TMT-B test, 17 drivers (39%) were still able to pass the MSDT.

On the descriptive level, only the MMST-results can be described to differ between those who failed and passed the MSDT with moderate significance (*p* = 0.035, U-Mann–Whitney) with an absolute difference of this mean at 0.5 points.

### Multivariable prediction models

A logistic regression relating the binary MSDT outcome to age, gender and all four cognitive test demonstrated strong evidence for age (*p* = 0.0008) and weak evidence for MMST (*p* = 0.042) to predict MSDT; no evidence was found for the other variables, see Table 1.

**Table 1:** Odds ratios (OR), 95%-CI, and test statistics of the explanatory variables in the logistic regression. Variable importance was assessed using the difference of the AIC of the full model (model 1) and a model leaving one variable out (∆ _(AIC)_); positive values indicate better model fit

	**OR (95%-CI)**	***z*-value**	***p*-value**	**Delta **_**(AIC)**_
Age	1.09 (1.04—1.15)	3.35	0.0008	11.60
MMST	0.81 (0.66—0.99)	-2.04	0.042	2.35
Gender	1.31 (0.53—3.27)	0.60	0.55	-1.64
UT	0.95 (0.75—1.20)	-0.43	0.67	-1.81
TMT-A	0.93 (0.43—2.01)	-0.19	0.85	-1.96
TMT-B	1.00 (0.48—2.14)	0.01	0.99	-2.00

Variable importance was checked by AIC. For better interpretability, we calculated Delta_(AIC)_, the difference in AIC between the full model (model 1) and the model leaving one variable out. The higher Delta _(AIC)_ the larger the variable importance with positive values for a better model-fit and negative values for a worse model-fit according to AIC (Table 1). Best model fit according to AIC was a model incorporating age and MMST (Model 2, see Table 2). Validating the model using the validation data (N_valid_ = 62) gave a sensitivity of 73% and a specificity of 61%.

**Table 2:** Model selection according to AIC included only two variables, age and MMST (model 2). Every addition year of age resulted in a 1.09-fold increase in the odds to fail the MSDT. Every additional point in the MMST score reduced the odds to fail the MSDT by 19%

	**OR (95%-CI)**	***z*-value**	***p*-value**
Age	1.09 (1.04—1.14)	3.35	0.0008
MMST	0.81 (0.66—0.97)	-2.25	0.024

With the overall aim to evaluate the predictive value of the four cognitive tests for the MSDT outcome, a third model (model 3) incorporated just these. Here, the validation resulted in a sensitivity of 62% and a specificity of 52%.

**Fig. 2:**
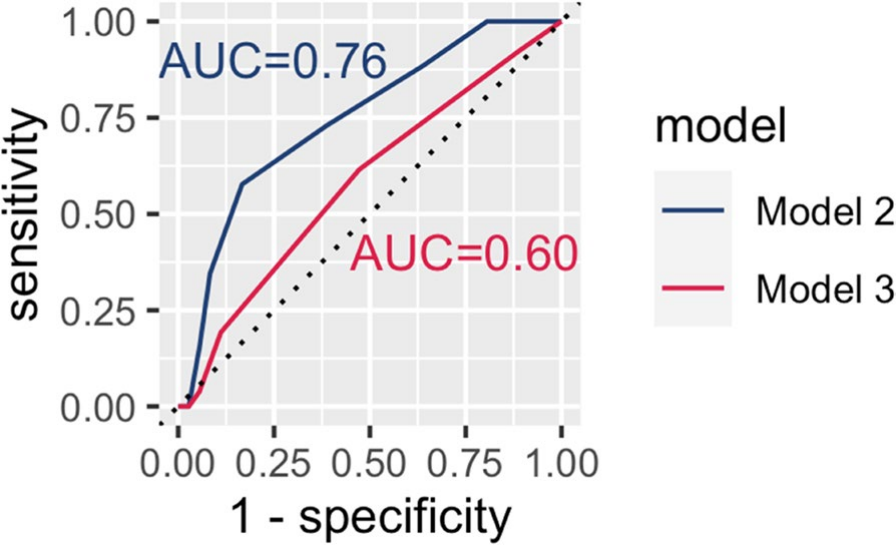
ROC and AUC of Model 2 and 3

We illustrated the inherent trade-off between sensitivity and specificity by adjusting cutoff values of probabilities resulting in ROC for model 2 and 3, (Fig. 2). AUC values can range from 0.5 (no predictive value) to 1 (perfect classifier).

As ROC, AUC and corresponding CIs potentially depend on the random 33%/66% split of complete data for training and validation subsets, we performed k = 2,000 iterations of random splits. After this, multivariable prediction models using age and MMST (model 2) yields a median AUC of 0.71 (95%-CI 0.57–0.85), while using four cognitive test results exclusively yields median AUC of 0.55 (95%-CI 0.40–0.71).

Using Model 2, the probability to belong to the class "failed" can be calculated using P_r_(Y = 1|X) = e^y^/(1 + e^y^) (inverse logit) with y =  − 0.491 + 0.083 ∗ Age − 0.219 ∗ MMST. For example, an 88-year-old patient with a MMST score of 17 has a probability of 95.7% to fail the MSDT.

## Discussion

Laboratory-based performance screens potentially offer to reliably predicting on-road performance of elderly drivers. Considerable efforts have been reported to evaluate the predictive power of individual test and combinations of individual test. If and how such validated screens might complement or replace the “gold standard” of on-road tests within a given country’s regulations might depend on critical evaluation of the actual system in place.

Thus, we here systematically evaluate predictive relations of the four standard cognitive tests currently in use in Swiss traffic medicine for MSDT outcomes, rather than establishing a novel toolset for prediction. Initial starting point was to confirm or falsify so far unsubstantiated rules of thumb partly persisting in practice. By establishing multivariable prediction models on a retrospective data set, we tested whether individual or combined test results allow predicting MSDT outcomes.

The logistic regression using all variables (model 1) finds age to be the single most dominant predictor for MSDT outcome with an Odds Ratio (OR) of 1.09 at a p of 0.0008. In other words, each year adds 9% chance to fail the MSDT. Model 2 includes MMST results as a meaningful predictor at an OR of 0.81 (*p* = 0.042). Validating model 2 yields a median AUC of 0.71 (95%-CI 0.57–0.85) and can be deemed of statistical value [20, 21]. Using four cognitive test MMST results exclusively (MMST, CT, TMT-A and TMT-B, model 3) yields a comparatively low median AUC of 0.55 (95%-CI 0.40–0.71). Thus, model 3 can be deemed of very little to no statistical value.

While the combination of age and MMST (model 2) might provide a broad orientation as to MSDT outcome with some validity, the cognitive tests results – alone or combined – cannot be used to predict impaired driving in older adults. Our analysis thus indicates that within the assessment for the fitness to drive of the elderly in Switzerland, there is very little to no evidence allowing “rule of thumb” for predicting MSDT outcome based on any of the four individual cognitive test or combinations thereof, alone.

The four standard tests studied here cover general cognition/mental ability (MMSE), attention and concentration (TMT-A), executive function (TMT-B) and visuospatial skills/construction (clock drawing) [22, 23]. Reger et al. [22], but also Mathias et al. [23], report these tests to be the most frequently used in the respective domain within their literature- or meta-analyses, the only exception being of the clock-drawing test.

As safe driving requires “the complex interaction of physical, cognitive, perceptual, and psychological skills and abilities” [24], there is a wide range of combinations, either focusing on cognitive abilities or also capturing vision, motor function and recorded or self-reported driving incidents [25–27]. Of the “cognitive” tests, each might focus on different domains, such as attention, construction/visuospatial skills, memory, executive function, perception or span general mental ability [22, 23, 28]. Furthermore, such studies, measures of “safe driving” might be, on the one hand, (odds ratios for subsequently recorded) motor vehicle crashes (MCV), study-associated tests of on-road driving performance or non-road (e.g. simulator-based) driving performance.

Meta-analyses and systematic reviews congregating statistical information form efforts to predict (on-road) driving performance come to varying conclusions: A 64-study review by Dickerson et al. [29] supports adapting the screening tool-sets to medical conditions, as a single tool is insufficient to determine fitness to drive. Along these lines, a meta-analysis of 27 reports by Reger et al. [22] does report overall significant relations between neurophysiological functioning and driving ability as measured by on-road tests and non-road-tests for adults with dementia. In contrast, Mathias and Lucas [23] evaluate 21 studies to compare, among others, the predictive values of a wide range of tests for on-road, simulator driving performance or crash history, carefully selecting for > 55-year old community drivers not diagnosed with dementia. The authors conclude that the predictive or discriminative ability of individual tests depended on the performance parameter (on-road, simulator or MCV), the exception being the UFOV, which identified as predictor of all three outcome paradigms and which emerges as a test with significant predictive value in other predictive toolboxes [25, 30–32].

For each individual test evaluated in this study, there exist mixed results on their predictive abilities (see below). Congregated analyses of individual such reports allow putting our individual results in a bigger context [22, 23, 29], although data sets diverge vastly either in size, outcome measure, combination of test, or prevalence of unsafe driving. This prompts justified efforts to compare a wide range of tests in forward-studies under the same conditions [33].

TMT-A and TMT-B turned out to be of surprisingly low predictive value in our study at OR of 0.93 (95%-CI = 0.43—2.01, *p* = 0.85) and OR of 1.00 (95%-CI = 0.48—2.14, *p* = 0.99) as compared to other studies. In [23], the TMT-B showed relatively large (dw = 0.79) and significant (0.63–0.95) differences between those who passed or failed an on-road assessment at an *N* = 195 from 3 studies suggesting a high degree of confidence. Other individual studies showed mixed results with respect to crash and performance outcome [25, 30, 31, 34, 35] for TMT-B. TMT-A tests showed very low differences (effect size dw = 0.21) and insignificant (0.08–0.34) differences between those who passed or failed an on-road assessment at an *N* = 230 from 3 studies in [23], and again mixed results from [36] and [35]. In contrast, our data show indiscriminate and non-significant levels of difference and large variation between those who passed or failed an on-road assessment for both TMT-A and TMT –B.

Our data indicate weakly significant differences in MMST results alone between those who passed or failed the MSDT, falling in line with largely diverging results from various studies ranging from evaluating MMSE as a (moderate) predictor [21, 25, 32, 36–38] or no or a very poor predictor [39–42] for safe driving in varying cohorts and conditions. Others highlight a differential MMST-subtest sensitivity in elderly drivers with and without cognitive impairment [28].

Overall, generally accepting an AUC of 0.7 – 0.9 as acceptable for a “good” predictor [20, 21], an AUC of 0.76 when combining age and MMST-result might be considered useful and applicable in practice. However, while a calculated likelihood of failing the MSDT as based on model 2 might be useful in deciding to grant an MSDT, we consider model 2 only to be useful for a broad and informational tool for orientation as to MSDT outcome.

As compared to other approaches trying to provide comprehensive and cost-effective tools to potentially complements or – wherever necessary– replace on-road performance tests, our analysis and resulting data are clearly neither intended nor by any means strong enough to replace MSDT. Large scale validation studies do show that such tools can reliably identify those elderly drivers with a high likelihood of failing on road tests, be it due to dementia [43], or due to (mild) cognitive or visual impairment [33]. In addition, off-road screening tools for safe driving in the elderly could be shown to benefit both time- and cost-wise from including also from simple non-cognitive information, such as number of medication taken per day, cervical spine mobility, impaired visual acuity or field of view and avoidance behavior, while maintaining high validity to results from on-road testing [26, 27, 44]. Effective screening tools might promise a possibility to alleviate cost and personnel related issues in relation to on-road tests, such as the MSDT.

The study had a number of limitations. It included a limited cases for a limited time spanning the years 2017–2019. As compared to other studies (eg. [33] with *N* = 560 or [42] with *N* = 17,538) a sample size of *N* = 225 is rather small, which might potentially limit the value of conclusions.

Additionally, but similar to other, prospective studies [21, 25, 28, 32, 38], cases were included on a non-random and retrospective basis, conferring an inherent selection bias. Factual MSDT and test data were only available and meaningful for cases in which cognitive tests or other information from either records or the traffic medical examination strongly recommended an MSDT, excluding substantially more severe and less severe cases. While it would be desirable to establish a large scale prospective study that relates cognitive test results to MSDT outcomes of volunteer drivers over a large range of ages and conditions, such a study is virtually impossible for practical, legal, organizational and financial reasons. In any case, this – or a conceivable prospective study – might still be confounded by additional conditions, e.g. somatic and psychological conditions not accounted for in the context of the specific aim.

The data sample can be criticized as based on some pitfalls in the current process in place of MSDTs in general. Here, the decision basis to allow for an MSDT is hardly accessible to full transparency, objective quantification and quality control so far. Similarly, the MSDT assessment and decision itself would benefit from harmonization and, potentially, a standardized scoring system. Separating staff allowing for an MSDT and performing it (blinding) would be an additional prerequisite for any unbiased result. Moreover, any inter-rater and inter-driver variabilities are complicated by variations in traffic and routes chosen for MSDTs. While certainly useful and desirable, attempts to tabulate and score route and traffic complexities [45], final MSDT outcomes are unlikely to become fully automated or fully objective/fair and will, thus, remain rater-dependent to some degree.

Based on the latter limitations, a potential strength of the study might be that it relates and limits findings to a specific aim and setting, in an analysis largely without involvement of the authors in any of the decisions with respect to granting and rating an MSDT. Conclusions are clearly limited to the objective to evaluate above-mentioned rules of thumb on this retrospective data set.

On the level of the multivariable prediction models, we approached missing data using a complete case (N_complete_ = 188) analysis rather than an imputation approach. Although exclusion of these 16% (N_ex, stat_ = 37) of all cases as incomplete might lead to additionally reduced statistical power, we believe that there are no systematic differences between the missing values and the observed values, i.e. that these are at complete random (MCAR) [46, 47]. We do however augment the training data, using SMOTE to balance class differences [48]. Both problems are unlikely to be solved per se by collecting more data in future pro- or retro-spective studies.

However, such future studies observing the statistical relations between medical and on-road assessments, i.e. screens and driving performance, would greatly benefit from more structured and numerical data in decision-making processes. Current efforts to simplify and harmonize MSDT evaluation-sheets might be complemented with standardized error counting (e.g. SAFE [49, 50] and establishing more transparently standardized MSDT-parcours. The latter might be established on (video-based) tracking and evaluation of road situations based on operational and tactic errors in relation to cognitive/executional domains.

## Conclusions

Our real life data indicate that, while being unquestionably useful indicators for cognitive impairments an thus, an MSDT, the limited standard set of cognitive tests alone or in combination, as currently used in traffic medicine examinations in Switzerland, are poor predictors of MSDT outcome. Only in conjunction with age MMST results might provide broad orientation for MSDT outcomes. Purported “rules of thumb” should not be applied on this basis.

With all limitations and caveats, our finding underscores the value of the MSDT for the elderly driver with borderline FTD and is reassuring for the integrative and multi-facetted approach to assess the FTD as provided by Swiss law. At the same time, our discussion identifies clear potential for improvement in the overall process.

## Data Availability

R-Code generated and/or used during the current study is available on OSF (see https://osf.io/fk4v2/). Data sets are available upon request.

## Abbreviations

AIC: Akaike Information Criterion
AUC: Area under the curve
CI: Confidence interval
CT: Clock test
EPP: Events per parameter
FTD: Fitness to drive
MMST: Mini mental status test
MMSE: Mini mental status exam
MMR: Mimum medical requirements
MSDT: Medically supervised driving test
OR: Odds ratio
RA: Representative of the authorities
ROC: Receiver operating characteristics
SMOTE: Synthetic minority oversampling technique
TME: Traffic medicin expert
TMT-A: Trail making test A
TMT-B: Trail making test B
